# Pearls before Swine: Plant-Derived Wastes to Produce Low-Cholesterol Meat from Farmed Pigs—A Bibliometric Analysis Combined to Meta-Analytic Studies

**DOI:** 10.3390/foods12030571

**Published:** 2023-01-28

**Authors:** Filippo Bertocci, Giuseppe Mannino

**Affiliations:** 1 Department of Veterinary Medicine and Animal Productions, University of Naples Federico II, 80134 Naples, Italy; 2 Department of Life Sciences and Systems Biology, University of Turin, Via Quarello 15/a, 10135 Turin, Italy

**Keywords:** agri-food waste, plant bioactive compounds, circular economy, sustainability, agricultural waste, polyphenols, sustainable farming, VosViewer, BiblioShyny, RevMan

## Abstract

Due to environmental and human factors, there is a growing amount of agri-food waste worldwide. The European Commission is incentivizing a zero-waste policy by 2025, pushing to find a “second life” for at least the avoidable ones. In this review, after summarizing the nutritional values of pork and the importance of its inclusion in human diet, a phylogenetic analysis was conducted to investigate potential differences in the structure and activity of HMGCR, which is a key enzyme in cholesterol metabolism. In addition, a bibliometric analysis combined with visual and meta-analytical studies on 1047 scientific articles was conducted to understand whether the inclusion of agro-food waste could affect the growth performance of pigs and reduce cholesterol levels in pork. Although some critical issues were highlighted, the overall data suggest a modern and positive interest in the reuse of agri-food waste as swine feed. However, although interesting and promising results have been reported in several experimental trials, further investigation is needed, since animal health and meat quality are often given marginal consideration.

## 1. Introduction

Food waste is a serious issue around the world due to the negative effects it has on the environment, economy, and society. Currently, the rise of food waste can result from both human and environmental factors. The first includes the world’s growing population, the actions of suppliers along the food chain, and the decisions of food service providers or consumers [1,2,3]. On the other hand, climate changes are the most challenging environmental factor, as it leads to an increase in non-marketable products [4,5,6].

In 2019, it was estimated that global food loss and waste was about ~1.3 billion tons per year, which corresponds to one-third of the food produced for human consumption. The Food Waste Index Report stated that ~931 million tons of food waste were generated in 2019, and Latin America and Europe ranked among the countries with the highest consumer waste rates, accounting for 200 and 180 kg per capita per year, respectively [7]. Although food waste in industrialized countries mainly occurs at later stages of the production chain, a considerable amount is also produced during earlier steps due to inadequate collection, storage and cooling methodologies. Moreover, according to the Food Waste Index Report, also developing countries produce a significant amount of waste (about 120 kg per capita per year) primarily because of the lack of technology for harvesting and storing fresh products [7,8,9]. Consequently, food waste is a global trouble, affecting all realities. For this reason, in recent years, several scientific studies have been conducted with the aim of searching for more sustainable waste management [10,11,12,13].

Agri-food waste is defined as the residues of agricultural products generated during both early and later production stages [14,15]. These residues include different and various plant materials, such as whole fruits, grains, roots, tubers, husks, stems, seeds, bran, germs, pomace, and pulp. Within them, roots, tubers and oil crops constitute the largest group, contributing 26% of the total production, which is followed by fruit-derived waste that contribute up to 22% [10]. As most agri-food waste is easily converted into other products, their excessive generation can be considered as a reflection of human behavior rather than food quality or edibility [16]. In addition, unlike other by-products, agri-food waste is rich in essential macronutrients (carbohydrates, proteins, lipids, dietary fiber, vitamins, etc.) and phytochemical compounds that can exert interesting biological functions [1,17,18,19,20]. Therefore, although not useful for human consumption, agri-food waste can be processed into edible ingredients for animal feeding [21].

In the last years, agricultural and food industrial byproducts has attracted the interest of researchers, legislators, industry, and consumers, also encouraging the European Union to promote producers toward a zero-waste economy by 2025 [22,23,24,25,26]. In this direction, recent scientific discoveries offer opportunities for the efficient reuse of food waste, leading to the development of innovative products of great economic importance with various experimental applications. For example, the use of agro-food waste has been recently evaluated (i) in plant sciences for the production of fertilizers [27,28,29] and biostimulants [30,31,32,33] as alternative to agrochemicals; (ii) in human sciences for the formulation of dietary supplements, fortified or functional foods [34,35,36]; and (iii) in animal sciences for the fortification of feedstuffs in aquaculture [15,37,38,39], aviculture [21,40,41] or livestock [42,43,44,45]. The use of agro-food waste as sustainable ingredients for the feeding of farm pigs is a topic that has recently attracted the interest not only of researchers but also of livestock companies. Indeed, the use of agro-food wastes would (i) significantly reduce feed production costs, (ii) solve the problem of disposing of wastes that cannot be used for human consumption, (iii) lead to the creation of functional feeds that can potentially influence the animal growth performance, and (iv) replace the use of drugs and antibiotics traditionally used to counteract the negative effects of the intensive livestock farming system [46,47,48].

In our recent work, we conducted a bibliometric investigation coupled with a meta-analytical analysis to evaluate the potential of agro-food waste on the growth performance of fish grown under controlled conditions in aquaculture systems [15]. Among the main results, it was shown how phytoconstituents still present in agro-food wastes can modulate the innate immune system and antioxidant defenses of fish, resulting in increased product quality. Here, with very similar aims, we analyze the potential effect of using agro-food waste as ingredients to supplement pig diets. Specifically, after describing the importance of the inclusion of pork in the human diet, we took an overview of the current global economic condition of pig farming, and we used a bibliometric approach coupled with VosViewer and BiblioShiny analysis [49,50] to examine the trend of scientific research related to the use of agri-food waste as a component of the swine diet [51].

## 2. Nutritional Value of Pork Meat

Pork is a rich source of high biological proteins, namely macromolecules having structure, quantity, and essential amino acid ratio comparable to human proteins. Consequently, unlike plant proteins, they are easily reusable during human metabolism [52,53]. Among these proteins, pork has been shown to be an interesting source of collagen, which is a protein mainly involved in counteracting aging processes and maintaining elastic and healthy skin [54,55]. In addition to proteins, many essential and functional amino acids can also be assumed almost exclusively through the consumption of pork meat [56,57]. These include (i) taurine, which is essential for infants and children; (ii) 4-hydroxyproline, which improves antioxidant function and prevents intestinal colitis; (iii) creatine, which is an antioxidant and an important component of energy metabolism in brain and skeletal muscle; (v) carnosine, and (vi) anserine, which plays a key role in protecting mammalian cells from oxidative stress [57]. Pork is also a rich source of minerals, particularly iron. Although other minerals such as potassium, sodium, phosphorus, zinc, and selenium are contained in pig meat, iron content is significantly higher than in most plant food. Moreover, unlike plant trivalent iron, swine iron is principally chelated by the EME group inside red blood cells, and it is highly bioavailable [58]. The vitamin content of pork is extremely specific and essentially consisting of B vitamins, namely cobalamin (vitamin B12), pyridoxine (vitamin B6), and thiamine (vitamin B1). In contrast, while antioxidant vitamins are present only in trace amounts, fat-soluble vitamins, such as retinol (vitamin A), ergocalciferol (vitamin D), phylloquinone (vitamin K1) and menaquinone (vitamin K2) can also be obtained with pork consumption [59]. Finally, while there is a certain variability due to the species, type of muscle, age, breeding and feeding of the animal, pork contains significant amounts of lipids, mainly triglycerides and cholesterol. As a result, it is considered not only a high-calorie food but also atherogenic and poorly digestible [58].

**Figure 1:**
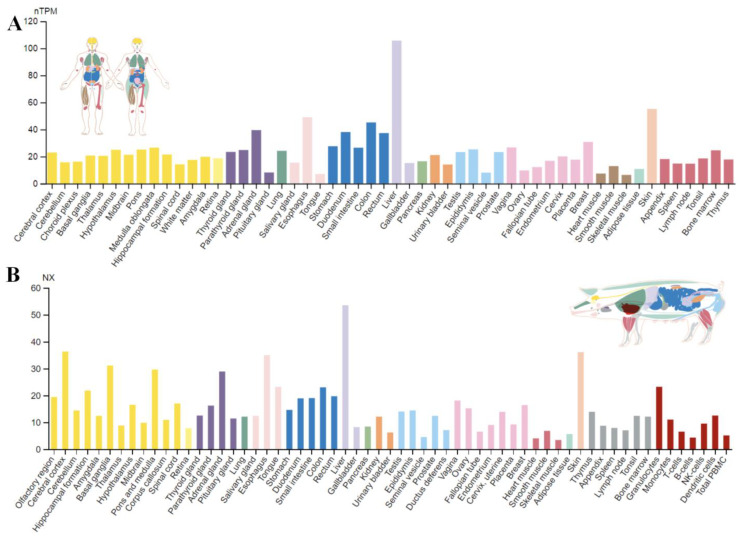
HMGCR expression profile in human (**A**) and pig (**B**) tissues. Data credit: The Human Protein Atlas [60,61] and The Pig RNA Atlas [61,62]. Data summary images were obtained from v22.proteinatlas.org via https://www.proteinatlas.org/ENSG00000113161-HMGCR/tissue (human protein expression profile, accessed on 10 January 2023) and https://www.rnaatlas.org/ENSSSCG00000014080-HMGCR (pig protein expression profile, accessed on 10 January 2023).

A major cause of increased cholesterol levels in pork is 3-hydroxy-3-methylglutaryl-coenzyme A reductase (HMGCR). Unlike humans, in which HMGCR gene transcription mainly occurs in the liver (Figure 1A), the swine gene is primarily transcribed in the ovaries and liver, but considerable levels can be also detected in the stomach, intestine, skin, and brain (Figure 1B). 

HMGCR is the enzyme controlling the rate of the mevalonate pathway, which is the metabolic way responsible for producing cholesterol and other isoprenoids. In particular, HMGCR catalyzes the conversion of HMG-CoA to mevalonic acid [63,64,65]. From a transcriptional point of view, the gene encoding for HMGCR shows a relationship not only with serum lipid traits but also with commercially quality traits, and it has always been considered an interesting candidate gene for assisted gene selection in pigs [66,67]. 

However, the different transcriptional site would not alone explain the high cholesterol levels characteristic of pork meat in comparison to other animals. Indeed, porcine HMGCR might be structurally similar but different for the activity. Accordingly, in order to investigate potential differences in protein sequence, a phylogenetic analysis was conducted. Specifically, using human HMGCR as the original entry, a database containing the amino acid sequence of the HMGCR protein currently sequenced in animals was constructed. The database was built using the BLASTp search of National Center for Biotechnology Information website (NCBI; https://www.nih.gov; accessed on 10 January 2023). Nonredundant (nr) protein sequences were chosen as queries, and the search was further restricted to organisms contained in specific libraries (taxid: 40674). The search algorithm was then adjusted by setting the threshold for the expected number of random matches in a random pattern (Expected Threshold) to 0.05 and the seed length that initiates an alignment (Word Size) to 6. Concerning the scoring parameters, the BLOSUM62 matrix was adopted, and the Gap Cost for the creation and extension of a gap in an alignment was set to 11 and 1, respectively. Then, all nr results having E values ≤ 1 × 10^−5^ were extracted through an iterative screening process. The putative amino acid sequences of the selected organisms were used to compute the distance tree of the results using NCBI TreeViewer (ver. 1.19.4) [68]. Phylogenetic tree visualization was performed through CLC software, and hierarchical protein classification was performed by the Neighbor Joining method, with a maximum difference of 0.85, and organized according to Grishin's view [68].

**Figure 2:**
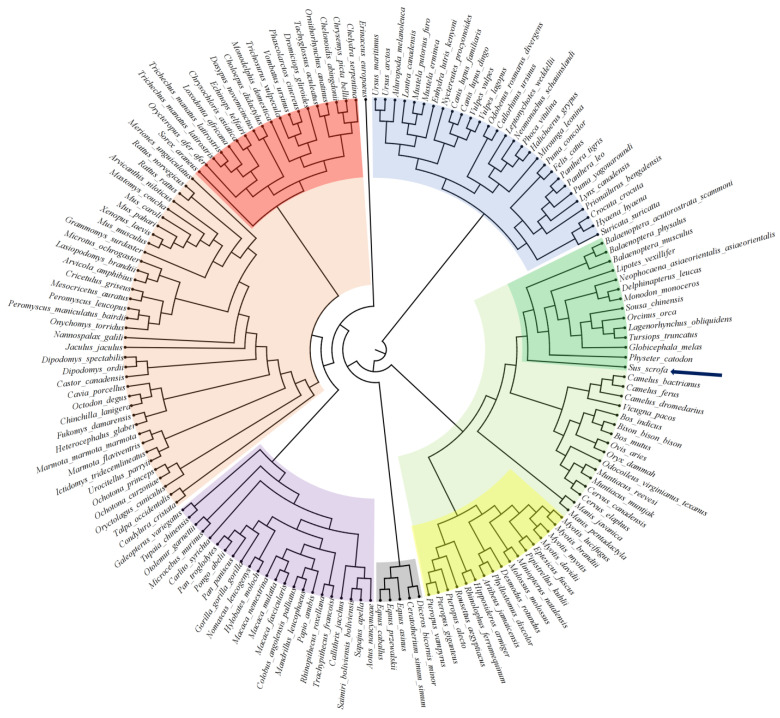
Phylogenetic analysis of 3-hydroxy-3-methylglutaryl coenzyme A (HMG-CoA) reductase (HMGCR) originated from amino acid differences in animal protein sequences. The circular tree was created by downloading amino acid sequences from NCBI using BLASTp search, while the distance of each entry was calculated using CLC software. In the figure, a division can be observed between carnivores (blue), whales and dolphins (dark green), even-numbered ungulates (light green), bats (yellow), odd-numbered ungulates (gray), primates (purple), rodents (orange) and marsupials, planktonic and oviparous (red).

The analysis allowed the clustering of HMGCR in animals according to protein sequence similarity degree. Specifically, five main clusters were delineated. The first group (blue shadow) contained carnivore animals. Among these, a clear distinction between felines and canines can be observed. The second cluster was composed of three smaller groups, namely whales and dolphins (dark green shadow), bats (yellow shadow), and even-toed ungulates (light green shadow). Interestingly, pigs (dark blue arrow) were in an intermediate position between whales/dolphins and even-toed ungulates. This apparently discordant finding is instead in line with both the hypothesis that sees Hindoyus as a common ancestor of cetaceans and pigs [69] and the high cholesterol content of aquatic animals, including sharks, dolphins, and whales [70]. The third group (gray shadow) is the smallest, and it includes only five species of odd-toed ungulates, among which the best known are horses. The fourth cluster (purple shadow) was composed of primates, which surprisingly did not demonstrate the highest similarity degree with the HMCGR protein. Finally, the last cluster is a very heterogeneous group, mainly consisting of rodents (orange shadow) and other animals, including placentals, oviparous, and turtles (red shadow) (Figure 2).

These minor structural differences could explain the distinct enzymatic activity observed between pigs and other mammals. Indeed, although the enzymatic reaction catalyzed by porcine HMGCR is totally equivalent compared with that observed in other animals, its enzymatic activity seems to increase with advancing age [71]. In contrast, it is well known that plasma lathosterol concentrations in humans and hepatic HMGCR activity in rats decreased rapidly after birth [71].

Despite the genetic and biochemical differences, many authors believe that pigs may be the most useful models for the investigation of hypocholesterolemic effects of drugs intended for human use [72,73]. Indeed, similar to humans, pigs are one of the few species that transport the majority of cholesterol in LDL. It has also been shown that pigs can develop atherosclerosis following hypercholesterolemic diets. Additionally, the plasma lipid profiles of ovariectomized pigs, males or females, have very similar trends to those observed in mans, females, and postmenopausal women, respectively [72,73].

## 3. Pig Breeding: Current Situation

Over the past century, the constant population growth has been associated with an increase in food demand [74,75,76]. Although consumer interest for healthy food has expanded due to the COVID-19 pandemic, only 5% of the world's population is today vegetarian, while 60% is flexitarian [75]. Among the European meat-consuming countries, Italy and Polonia reported fairly moderate consumption (about 50% of the total consumers), while Spain (65%), Germany (70%), France (75%), and the United Kingdom (80%) have drastically higher percentages [77] (Figure S1).

Within foods of animal origin, pork is the most consumed in Europe and Asia, and it is the second worldwide after chicken [77]. Although it is well known that the over-consumption of pork is associated with the emergence of harmful physiological complications [63,78,79,80,81], its success comes from the relatively low cost and the high nutritional values [58]. An additional problem involving pork is related to the susceptibility to lipid oxidation, especially when it is minced. Microbial contamination, along with lipid oxidation, is the main factor in meat degeneration. Accordingly, the foodborne pathogens *E. coli*, *S. aureus*, *Salmonella* spp. and *Listeria* spp. are regularly found in meat products [80,82,83].

Pig breeding probably began during the Neolithic era, when humans developed sedimentary traits and began farming animals unable to practice transhumance, including pigs. Conversely, their domestication occurred in China between 8000 and 5000 B.C., and unlike the modern era, they were grown in the wild. The practice of barn raising only started in the late Middle Ages. Until the 1960s, pig farming was mainly based on several traditional family and small-scale farming systems, which typically consisted of 4000 m^2^ of pasture for about 20 pigs. However, in the second half of the 20th century, this farming style became intensive, leading to a large increase in pig production [84]. Today, the largest pig production company in the world is located in China, raising 1.1 million breeding pig sows [79], while the main pig-exporting European countries are the Netherlands and Denmark, while Canada exports to the United States. As the world's third largest producer and consumer of pork, the United States plays also a significant role in the economy, with exports accounting for 20% of the global commercial production [85].

Although intensive farming is an important food source for human needs, it is a challenging condition for swine. Indeed, the stress caused by a stimulus-free environment leads animals to become aggressive, resulting in mutual scratching and biting that increases the risk of infection and disease. In addition, the extreme conditions of intensive farming push pigs beyond their physiological capabilities, exposing them to lowering of immune defenses [86]. Consequently, farms preventively administrate antibiotics as prophylaxis, but their exponential use has a negative impact on both human and animal health, because it contributes to the development of antibiotic-resistant bacteria [86].

Conversely to this scenario, the modern consumer has raised the interest toward animal-derived foods obtained through sustainable farming systems, which are perceived as less harmful for human health [48,87]. Accordingly, since agri-food waste matrices are rich in phytoconstituents also exerting potential antibacterial and antiviral activities, it could be a sustainable strategy for pig farming not only to reduce the costs of organic waste disposal but also an alternative to decrease the excessive use of antibiotics [87]. However, can this practice be a sustainable way in terms of food quality and safety? To answer this question, a bibliometric study coupled with visualization analysis was conducted to understand the degree to which this topic is addressed in the scientific literature. In addition, in order to assess the potential positive and/or negative effects of using agro-food waste as an ingredient in pig diets, a meta-analysis study of data collected from articles published in the past 15 years was conducted.

## 4. Bibliometric Analysis

Bibliometrics is a branch of scientometrics, belonging to information sciences, which aims to apply qualitative and quantitative approaches to academic publication [88,89]. It is widely used to examine results derived from a set of bibliographic records to construct theories. Specifically, it (i) summarizes research trends over a time frame; (ii) highlights interest for a specific research field; (iii) identifies nations, affiliations, authors, and entities that contribute to a specific research topic; (iv) defines collaborations among nations, affiliations, authors, and entities; (v) provides a comparative analysis of scientific productivity at the country, province, city, institution, or individual level; and (vi) understands how advanced the research topic has become within specific social realities [88,89].

**Figure 3:**
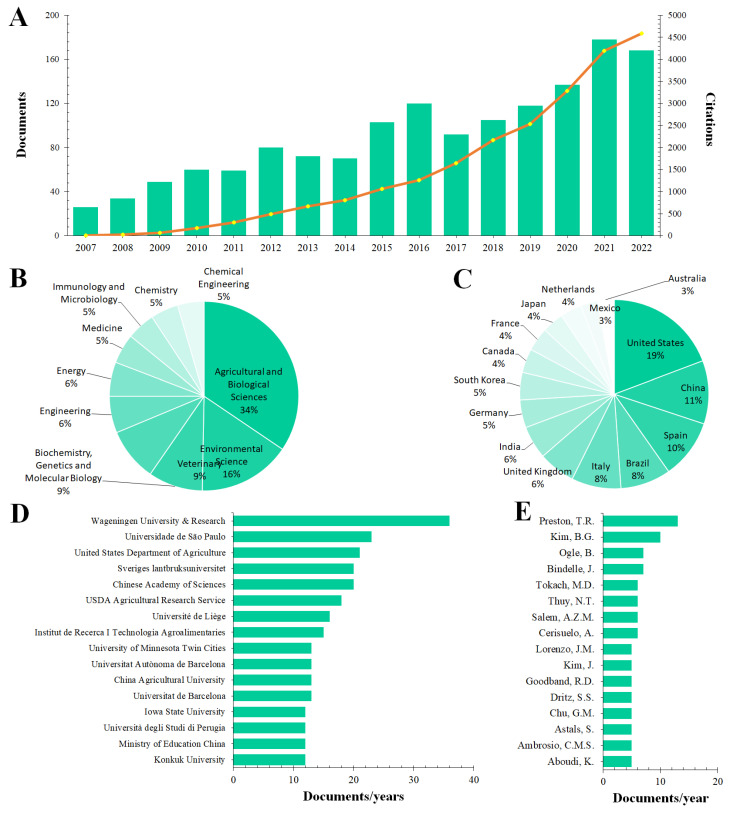
Bibliometric results related to the use of plant waste for swine feeding. (**A**) shows the number of scientific articles published between 2007 and 2022; (**B**) shows the top 10 scientific areas; (**C**) shows the top 15 countries with the most articles; (**D**) shows the top 15 affiliations with the most articles; and Panel (**E**) shows the authors with the most publications.

In this work, bibliometric analysis was used to summarize the current situation related to the use of plant-based food waste for supplementing a farmed pig diet (Figure 3). 

In particular, with the aim of creating a database comprising recent scientific publications, bibliometric data were retrieved from different scientific search engines, including Scopus, Google Scholar, ISI Web of Knowledge, and PubMed. The following keyword search string was manipulated to collect potential scientific publications: *TITLE-ABS-KEY (“disposal” OR “agri-food” OR “agrifood waste” OR “agricultural waste” OR “plant-based waste” OR “plant waste” OR “byproducts” OR “by-products”) AND TITLE-ABS-KEY (“swine” OR “pig” OR “pork”) AND TITLE-ABS-KEY (“diet supplementation” OR “ diet inclusion” OR “dietary supplementation” OR “dietary inclusion”).* Moreover, the following string *(EXCLUDE “human” [All Field] OR “sheep” [All Field] OR “fish” [All Field] OR “chicken” [All Field] OR “rabbit”[All Field] OR “mouse”[All Field] OR “mice”[All Field])* was added to better target the literature search on the topic of interest. Punctuation, marks, and singular/plural forms were ignored, and the search was limited to articles published in the last 15 years (2007 to 2022, with a last check on 12 October 2022) and written in English. Precisely, 1645 entries were retrieved from the database and manually inspected. Accordingly, 571 articles were removed from the database. Finally, the title, abstract, keywords, and affiliation information of each selected article was downloaded in comma-separated values (.csv) format and used for bibliometric evaluations (Figure 3). 

Analyzing the 1074 articles related to “inclusion of agro-food waste in pig diets”, an increasing trend in the number of publications over the past 15 years was shown, reaching a peak of 911 and 751 articles in 2020 and 2021, respectively (Figure 3A). Precisely, nearly 50% of the articles were published in the last 5 years (2017–2022), and 75% of the total citations were recorded in the same years (Figure 3A). This result suggests a constant growing interest in the reuse of plant waste to supplement the diet of farmed pigs in the recent years, since the number of publications usually measures productivity, while the number of citations measures influence.

Regarding scientific fields, it is not surprising that the bibliometric analysis revealed Agricultural and Biological Sciences and Environmental Sciences as the most productive areas, accounting for 34% and 16% of the total published manuscripts, respectively (Figure 3B). However, the absence of the Food Science sector along with the low percentage for Veterinary (9%) and Medicine (5%) is alarming, because it would suggest a greater interest in researching plant disposal alternatives than in monitoring animal welfare or assessing suitability for food production for human consumption.

On the other hand, Figure 3C shows the countries that mainly contributed to the publications related to the use of plant waste as an additive in swine diets. The analysis revealed that the USA leads among countries, accounting for 19% of the total publications. It is followed by China, Spain, Brazil, and Italy that recorded a very similar percentage, ranging between 8% and 11%. This is a clear indication suggesting a strong attraction of European marketing researchers for this research topic, while American interest is more secondary. Indeed, when considering together the publication rates of the Netherlands, France, Germany, England, Italy and Spain, it amounts to nearly 40%. Moreover, although the Netherlands was involved in only 4% of published articles (Figure 3C), the affiliation accounting for 35 publications per year across the different 185 institution was Wageningen University & Research (Figure 3D). Among the other affiliations, four were American (United State of Department of Agriculture, USADA Agriculture Research, Iowa State University and University of Minnesota Twin City), two were Chinese (Chinese Academy of Sciences and China Agricultural University), one was Spanish (Institut de Recerca i Tecnologia Alimentàries), one was Brazilian (Universidade de São Paulo) and one was Italian (University of Perugia) (Figure 3D). This result agrees with Figure 3C. Regarding authors, T.R. Preston (Scopus ID: 55067842000) and B.G. Kim (Scopus ID: 26654167200) have been identified as the most productive authors in the past 15 years (Figure 3E). However, although Kim has significantly fewer articles, he has only slightly fewer citations than Preston, and his h-index is currently four points higher than that of Preston.

## 5. Graphical Analysis

The database containing the 1074 scientific articles was also used for visual analysis using VosViewer and BiblioShiny. VOSviewer is a software for bibliometric analysis that allows the visualization of pre-existing networks between different articles. These networks can be constructed based on several factors, including citations, bibliographic pairing, co-citations, or co-authorship. Additionally, the software is able to perform text mining to visualize co-occurrence networks of terms and keywords from a corpus of scholarly literature [90,91]. Finally, VosViewer also allows the inclusion of publication year as an additional variable, achieving in the generation of maps describing the trend of the research topic over years [91]. On the other hand, BiblioShiny is an open-source tool designed to perform a comprehensive analysis of scholarly literature mapping. This software, coupled with other bibliometric analyses, provides a comprehensive and global view of existing networks between countries, authors, and research topics [50,92].

### 5.1. VosViewer Reveals Changes in Interest over Time for Both Countries and Research Areas

**Figure 4:**
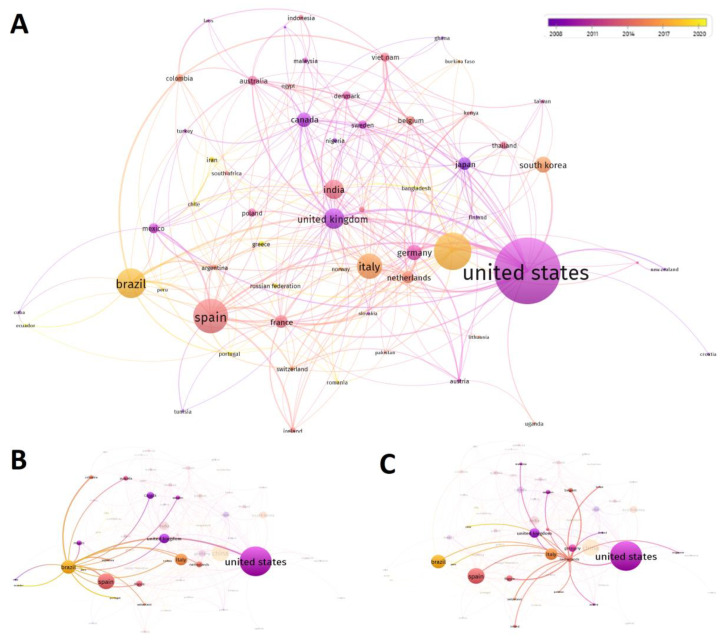
Co-authorship map showing the existing networks between countries involved in the publication of scientific articles related to the inclusion of agro-food waste in pig diets. (**A**) shows the global network of countries. (**B**) focuses on Brazil, and (**C**) focuses on the Netherlands. The size of the nodes indicates their frequency. The curves between the nodes represent co-occurrence in the same publication.

The previous database containing 1074 articles was then processed by VosViewer with the aim of visualizing a co-authorship map (Figure 4).

Since in some articles, the number of authors was found to be excessive (>7), the full author count method was applied for the analysis, considering each entry as a single weight, and not fractionated by the number of total authors. In addition, to avoid potential bias, articles in which the authors had more than one affiliation country were removed as well as those in which more than five different countries were reported in the affiliation section. Finally, exclusively entries having a fixed minimum number of document (n = 5) and citation (n = 10) were considered. Consequently, of the 119 countries, only 59 meet the above-mentioned thresholds, and the total link strength was calculated by considering documents, citations, interaction number, and frequency of subsisting interactions between different countries (Figure 4A). VosViewer analysis partially confirmed the data shown in Figure 3C, reporting the USA as the leading producer of scientific articles related to the research topic. The USA was followed by China and Brazil, along with three European countries, namely Italy, Spain, and the United Kingdom (Figure 4A). Again, the Netherlands showed lower production efficiency than other countries. However, considering the publication year of the analyzed articles, most of the U.S. publications were dated between 2008 and 2011. The United Kingdom also showed a similar trend as the United States. On the other hand, while Spain showed the most publications in 2014, Brazil and China recorded their peak between 2017 and 2022, demonstrating a recent growing interest for the research topic (Figure 4A). Regarding the collaboration network, Brazil had a strong relationship with European countries currently researching in this field, such as Spain, France, Germany, and Italy (Figure 4B). Moreover, the Netherlands recorded the highest number of interaction with all other countries, as demonstrated by the strong link shown in Figure 4C. This finding could explain the data reported in Figure 3D.

**Figure 5:**
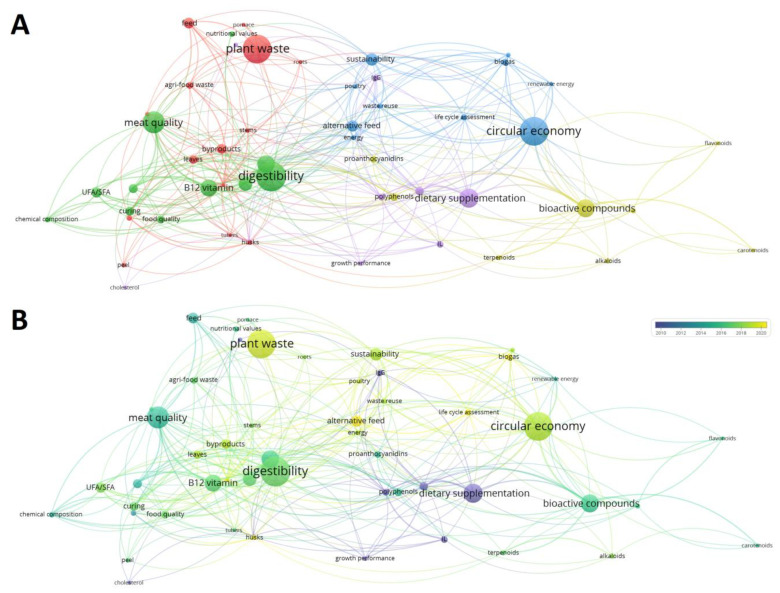
Co-occurrence map showing the existing networking of terms reported as keywords in publications related to the inclusion of agri-food waste in swine diets. (**A**) shows clustering in five different groups. (**B**) shows the same network from a temporal perspective. The size of the nodes indicates their frequency. The curves between nodes represent their co-occurrence in the same publication.

Regarding the keywords used in the analyzed scientific articles, a co-occurrence network map was constructed by exclusively considering the terms that were repeated at least 10 times in the relative section. In addition, plural/singular forms, punctuation marks, and/or synonyms were adjusted and manually unified. A total of 54 keywords having 321 different links were identified and plotted (Figure 5).

VOSviewer analysis allowed the division of the terms into five different clusters. Cluster I (Figure 5A, red dots) contained 14 items sharing the waste topic. Indeed, the most popular word was ‘plant waste’ (total link strength: 18; total links generated 14; occurrence: 68), immediately followed by ‘byproducts’ (total link strength: 10; total links generated 9; occurrence: 23). Specifically, several agricultural waste were identified, including leaves, piglets, roots, and stems. Cluster II (Figure 5A, green dots) contained 11 items, mostly related to ‘food quality’, ‘meat quality’, and ‘digestibility’. These terms appeared about 70 times in the different papers and had a strong connection with each other. Other terms included in cluster II were related to ‘chemical composition’ and to ‘nutritional values’ of meat products. Cluster III (Figure 5A, blue dots) contained 10 items. All these words were related to the alternative and sustainable use of agri-food waste for the production of ‘biogas’ (total link strength: 54; total links generated 21; occurrence: 68), ‘biomass (total link strength: 15; total links generated 11; occurrence: 25), or ‘renewable energy’ (total link strength: 10; total links generated 10; occurrence: 18). Interestingly, the word ‘alternative feed’ (total link strength: 15; total links generated 22; occurrence: 27) also appeared in the displayed network.

On the other hand, cluster IV (Figure 5A, yellow dots) included nine items, in which bioactive components typically found in plant matrices and agricultural wastes were listed. Among the various terms, ‘terpenoids’ (total link strength: 6; total links generated 11; occurrence: 12), ‘polyphenols’ (total link strength: 15; total links generated 22; occurrence: 19), ‘anthocyanins’ (total link strength: 9; total links generated 15; occurrence: 19), ‘proanthocyanidins’ (total link strength: 11; total links generated 13; occurrence: 26), and ‘alkaloids’ (total link strength: 6; total links generated 8; occurrence: 12) were found. However, the low frequency of these terms in the analyzed articles may suggest a negligence tendency in analyzing the phytochemical component of plant waste material. Finally, cluster V (Figure 5A, violet dots) included nine items, which were mostly attributable to parameters normally monitored during the growth of livestock animals, such as ‘growth performance’, ‘lipid panel’, and 'blood parameters’. From a temporal point of view (Figure 5B), the most frequently used terms were those related to the reuse of ‘plant waste’ (average publication year: 2019) for the production of ‘biomass’ (average publication year: 2018), ‘biogas’ (average publication year: 2017) or ‘animal feed’ (average publication year: 2020) under a ‘circular economy’ perspective (average publication year: 2021). This network would suggest a greater interest in sustainable processes and an awareness of the huge problem associated with the disposal of waste.

### 5.2. BiblioShiny Reveals the Usual Country–Topic–Research Field Linkages

**Figure 6:**
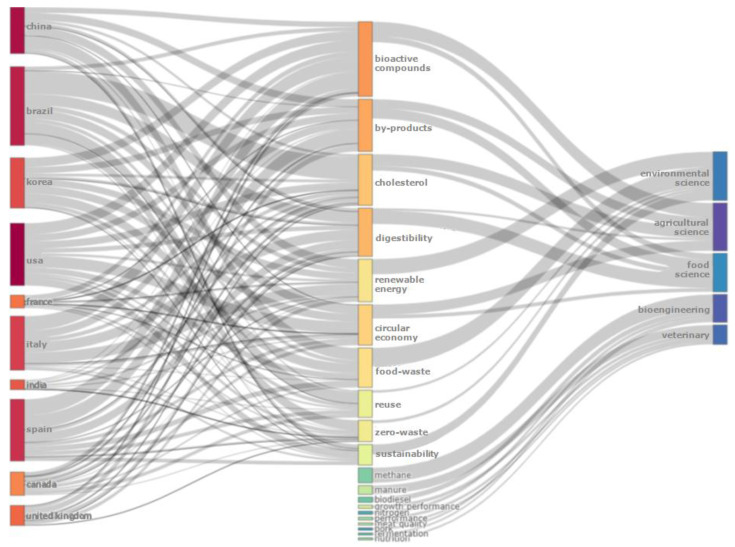
Three-field tree plot showing the existing connection between the main countries contributing to the search topic, the most used keywords in the articles, and the main research areas. The three semantic areas are connected by a gray plot. The box size beside the terms indicate the frequency in publications, while the different coloring indicates the frequency of connections (red: higher frequency; blue: lower frequency).

BiblioShiny provides the definitive summary of the current scientific condition. Indeed, the software is able to create three-field tree plot in which different semantic fields are connected by a gray plot [50,92]. Figure 6 shows a chart in which the connection between (i) country contributing to the research topic, (ii) the list of most used keywords, and (iii) the main scientific fields is studied. The box size beside the terms indicates the frequency in publications, while the different coloring indicates the frequency of connections.

Ten main countries publishing articles on the topic of sustainable reuse of agri-food waste can be identified (Figure 6). Most of the countries were in accordance with data shown in Figure 3, Figure 4 and Figure 5. However, unlike previous analyses, in Figure 6, each country was also associated with the main keywords listed in the second branch. These connections provide useful information on the main research lines toward which each country is currently focusing its research. In particular, the USA and Brazil are the most active countries, but meanwhile, the USA seems to be indiscriminately interested in almost all topics, while Brazil is mostly focused on circular economy and sustainability topics. In contrast, it seems that other countries, such as China and Korea, focus more on reusing agro-food waste simply because it is rich in bioactive compounds rather than worrying about its disposal. Finally, Italy found several connections with the sectors listed in the third branch as “Food Science” and “Veterinary Science”, suggesting an interest in the reuse of food waste without harming the health status of humans and animals.

## 6. Meta-Analysis

Forest diagrams are a useful tool for meta-analytic studies to answer a common question because they summarize data from multiple papers in a single image. Specifically, all studies investigating the same question are identified and categorized in a forest plot and, after extraction of experimental data, a common statistic is applied for all the different entries. This process allows both the direct comparison of what different studies are demonstrating and the quality of the related results from a single diagram [51,93]. In the following subsections, our goal is understand whether the inclusion of plant wastes in pig diets can have positive effects on both animal growth performance and the cholesterol content of meat derived from their processing.

### 6.1. Growth Performances

The occurrence of diseases, lesions, and infections are major problems in pig farming and often result from the stress factors caused by the intensive farming methods [86]. The unregulated and uncontrolled use of antibiotics as prophylaxis will ultimately pose an additional problem for human and animal health, as the drugs normally persist in meat and tend to develop antibiotic-resistant strains [94]. Therefore, the use of feeds consisting of plant wastes could be a safer and cheaper alternative for disease prevention in pig farming also from a circular economy point of view. Indeed, in addition to being rich in macro- and micro-nutrients, plants and their derived wastes are rich in bioactive compounds that can act as antioxidants [15,37,45,87,95,96,97], antibiotics [98,99,100,101,102,103,104], antivirals [98,101,105,106,107], or growth promoters [108]. In this regard, many studies have shown that plant waste-based feeds can be effectively used as appetite stimulators in a variety of animals, including pigs [108,109]. Moreover, since feed accounts for 60–70% of the total cost of meat in the swine sector, the use of plant waste as an ingredient in livestock diets could indirectly reduce the cost in the market. In addition, the current increase in feed prices is one of the main obstacles to expanding local pork production, as locally produced meat has to compete with cheaper imported meat. Consequently, the use of unconventional feed resources that are safe for human and animal health could be an option in line with modern European guidelines [23,25,26,47,110].

**Figure 7:**
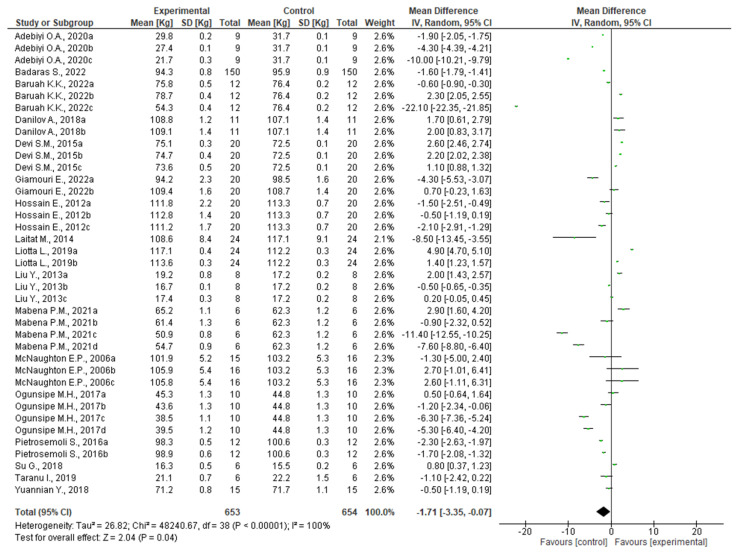
Forest diagram representation of growth performance of swine fed a diet inclusive of plant waste. Data were extracted from 17 articles [111,112,113,114,115,116,117,118,119,120,121,122,123,124,125,126,127] and plotted against the mean difference. Each horizontal line in the graph represents an individual study, with the experimental mean value displayed as a green box. For each study, if the horizontal line (95% CI) crosses the vertical line, no significant differences can be observed between experimental and control groups. The black diamond at the bottom of the forest plot represents the average effect size calculated from the combination of the results from all selected studies.

In order to test whether a diet enriched with plant waste could increase the growth performance of pigs, the search string used for the collection of articles in the previous section was supplemented with the following terms: *TITLE/ABS (“growth performance” OR “production yield” OR “growth promoters”)*. The additional string allowed the reduction in the database to 125 entries, which were manually screened by reading the title, abstract, and/or full text. Specifically, original articles were eligible for meta-analysis if they met the following criteria: (i) English had to be the language used for writing the article; (ii) only entries from peer-reviewed journals were considered; and (iii) scientific experts were required to have evaluated the manuscript prior to publication. In addition, for this type of analysis, (iv) only randomized and controlled clinical trials involving pigs from farm system were considered; (v) the intervention was supposed to supplement pig diets with plant wastes; (vi) the number of subjects used in the clinical trial should be clearly reported, along with the measured experimental data (mean and standard deviation or standard error); (vii) the experimental parameter evaluated in the article should be growth performance; (viii) when experiments included the use of different concentrations of plant wastes for feed formulation, all diets were individually processed as stand-alone entries; and (ix) if articles reported experimentation using different plant wastes incorporated in separate feeds, these were considered as separate entries. Finally, (x) only articles in which the experimental intervention was compared to control groups fed a normal diet could be considered. As a result, of the 125 published full-text articles, 108 were excluded. Data (*n* = 39) from the selected articles (*n* = 17) [111,112,113,114,115,116,117,118,119,120,121,122,123,124,125,126,127] were used for meta-analysis by constructing the forest plot diagram. Because the data were accumulated from a series of independently performed studies, all selected studies were not functionally equivalent. Consequently, the forest plot originated was obtained using the random effect based on the calculated heterogeneity among the studies. The statistical heterogeneity between studies was tested using Cochrane's Q test (significance level *p* < 0.05) and the I^2^ statistic. In addition, a sensitivity analysis was performed to verify the influence of each study on the overall effect size, and the potential publication bias was examined by visual inspection of the respective funnel plots. The combined results of selected articles from the random-effects model suggested a negative effect on pig growth performance after the inclusion of plant waste as an ingredient in feeds (WMD: −1.71; 95% CI: −3.35; −0.07; I^2^ = 100%; *p* = < 0.00001) (Figure 7). Specifically, 15 trials reported a decrease in pig weight following the use of plant waste in the diet [112,116,118,119,120,122,123,124,126], while 9 suggested a positive effect [112,113,114,119,120,121,125,126,127], and the remaining 15 showed no statistically significant (*p* > 0.05) effects [111,113,115,116,117,118,119,120,126]. However, the latter result can be positively interpreted, because pigs that did not record a decrease in weight had continued to normally increase their weight without exhibiting toxic or side effects. At the same time, agro-waste was successfully removed from the environment, achieving a money-saving effect for its disposal [37].

Regarding experiments reporting adverse effects on pig weight, in most cases, the entries introduced for forest plot analysis were derived from an experimental trial in which a dose–response effect was evaluated. For example, Baruah and colleagues investigated the potential from replacing cornmeal in the diet of pigs with dried banana seudostem powder at different rates [120]. The authors set up the experimental trial by testing inclusion percentages ranging between 5% (*w*/*w*) and 30% (*w*/*w*) (experimental group) and comparing the results with those obtained from pigs fed entirely cornmeal (control group). The authors, after verifying that all experimental diets were isonitrogenic and isocaloric, reported an increase in total body weight and average daily gain in pigs fed 20% (*w*/*w*) banana pseudostem compared to the other groups. However, pig performance was significantly lower when dietary levels of banana pseudostem were increased up to 30% (*w*/*w*). The authors concluded by stating that a maximum of 20% (*w*/*w*) banana pseudostem can be included in the diet of pigs.

Similar results were obtained in the study by Mabena (2021), who fed farm pigs a diet containing different percentages of amarula nut cake [119]. Following a very similar experimental protocol as Buruah, Mebena and colleagues replaced part of the cornmeal with vegetable waste ranging between 5% (*w*/*w*) and 20% (*w*/*w*). Again, the authors observed a dose-dependent effect, showing a strong reduction in pig weight when vegetable waste was included in doses greater than 150 g/kg. Moreover, when high levels of waste were included in pig diet, also protein digestibility and fiber levels were negatively affected. Therefore, the authors concluded that amarula nut cake could be included in pig diets only at less than 15% (*w*/*w*).

However, it is not always the best choice to replace cornmeal with plant waste in order to improve pig growth. Indeed, in the study conducted by Adebiyi, the possibility of including watermelon waste was evaluated, but it exclusively led to negative effects on growth performance [124]. In this case, the inclusion rates were much higher than in previously described studies, ranging from 20% (*w*/*w*) to 60% (*w*/*w*). However, considering the data obtained on the quality of meat derived from pigs fed watermelon waste, the authors do not discourage the use of this waste for pig farming, as it allowed the production of food that was qualitatively comparable to control meat.

In conclusion, this analysis suggests that although plant efficacy depends on multiple factors including dose, duration and route of administration, the most important factor is the percentage of waste inclusion in the feed. In addition, although many studies have shown how the inclusion of plant waste can have positive or negative effects on animal growth performance, very few studies have proven the influence on the general health of pigs. Indeed, the physiological, histological and digestibility parameters of plant waste have been only poorly investigated. Therefore, further studies on this aspect are needed.

### 6.2. Cholesterol Values

As a result of the growing market trend toward healthier foods, pigs have been subjected to very selective breeding over the past decade. In particular, the amount of intramuscular fat and its quality composition have an important effect not only on consumer preferences but also on the perception of meat quality and its economic value [128,129].

In addition to genetic selection, a potential approach to obtaining pork meeting the standards required by consumers could be to control the diet of breeding pigs to ensure reduced cholesterol levels. In recent years, research on dietary supplements has shown that plant extracts can effectively reduce blood levels of cholesterol in humans by acting on several biomolecular and biochemical targets, including HMGCR [63,130]. In this context, the action of botanicals is attributed to the presence of bioactive compounds that are naturally synthesized by plants and exert a variety of biological actions, including lowering cholesterol [131]. Consequently, a reasonable assumption is that the inclusion of plant wastes in pig feed can demonstrate additional effects to those on growth performance. Indeed, although plant wastes are considered unsuitable for human consumption, they still remain a rich source of secondary metabolites exhibiting important biological actions [46,132].

**Figure 8:**
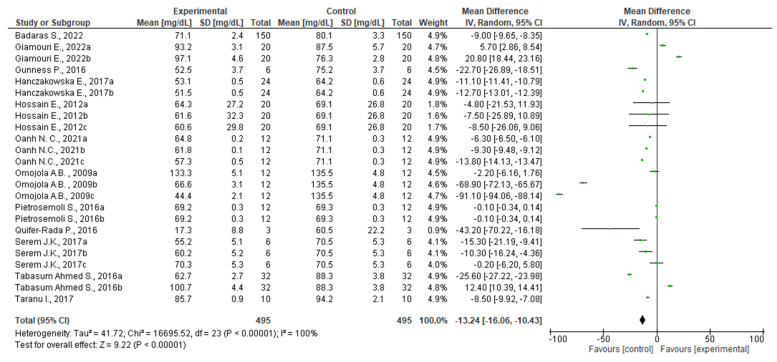
Forest diagram representation of cholesterol level of swine fed a diet inclusive of plant waste. Data were extracted from 17 articles [123,125,126,127,133,134,135,136,137,138,139,140] and plotted against the mean difference. Each horizontal line in the graph represents an individual study with the experimental mean value displayed as a green box. For each study, if the horizontal line (95% CI) crosses the vertical line, no significant differences can be observed between experimental and control groups. The black diamond at the bottom of the forest plot represents the average effect size calculated from the combination of the results from all selected studies.

In order to evaluate this hypothesis, a meta-analytic study was conducted using the same inclusion criteria described in the previous section. However, the search string was replaced with TITLE/ABS (“cholesterol” OR “hypo-*” OR “hyper-*” OR “blood parameter”) in order to allow the exclusion of articles not related to the research topic. As a result, of the 125 published full-text articles, 113 had been removed. Data (n = 24) from the selected (n = 12) articles [123,125,126,127,133,134,135,136,137,138,139,140] were used for meta-analysis by constructing forest diagrams. Because the data were accumulated from a number of independent studies, all selected studies were not functionally equivalent. Consequently, the forest diagram was obtained using the random effect, which was based on the calculated heterogeneity among the studies. Again, statistical heterogeneity between studies was tested using Cochrane's Q test (significance level *p* < 0.05) and the I2 statistic. In addition, a sensitivity analysis was performed to check the influence of each study on the overall effect size, and potential publication bias was examined by visual inspection of the respective funnel plots. The combined results of the selected articles from the random-effects model suggested a positive effect, reducing cholesterol levels in farm pigs after the inclusion of vegetable waste in their diets (WMD: −13.24; 95% CI: −16.06; −10.43; I^2^ = 100%; *p* = < 0.00001) (Figure 8). Specifically, 14 studies reported a strong decrease in cholesterol content [133,134,135,136,137,138,139,140], three suggested an increment of cholesterol levels [125,134], and the remaining seven showed no statistical (*p* > 0.05) effect [126,127,135,137].

Contrary to the observations for growth performance (Figure 7), the inclusion of agri-food waste achieved more concordant results, suggesting a strong effect on the reduction in cholesterol levels in farmed pigs. Among the clinical trials reporting negative effects on cholesterol levels, Tabasum Ahmed and colleagues have conducted an interventional study in which the effects of a herb mixture containing pomegranate, Ginkgo biloba, and licorice was tested in both natural form and following fermentation [134]. Although the authors demonstrated positive effects on the feed intake, back fat thickness of pigs, and lean production for both formulations, only the fermented mixture was able to effectively reduce cholesterol levels in pigs [134]. This result might suggest that in order to exert the desired effect in clinical trials involving animals, the agri-food waste should be further processed before dosing.

The clinical trial reporting the best effect on cholesterol level content in pork meat following the inclusion of agri-food waste is documented by Omojola and colleagues [137]. In this intervention study, the inclusion of garlic waste in pig diets was investigated, evaluating not only the cholesterol level but also the physical and sensory properties of pork. The complex clinical trial consisted of 48 Large White pigs randomly assigned to four dietary groups under a completely randomized design. Again, the plant matrix was processed before inclusion in the animal diet. Specifically, scraps of garlic bulbs were separated into cloves, sun-dried, ground, and then incorporated into the ration in order to replace a fraction of cassava meal. The trials involved the inclusion of different percentages of plant matrix, ranging between 0.50% (*w*/*w*) and 1.50% (*w*/*w*), were compared with control groups in which garlic was not included. Results showed a progressive reduction in total cholesterol level from 135.35 mg/100 g in the control diet to 44.44 mg/100 g in the diet with garlic supplementation at 1.50% (*w*/*w*). Other parameters, related for example to back fat thickness, also decreased with increasing levels of garlic in the diet. However, sensory analyses reported by the panelists revealed that pork from pigs treated with 0% (*w*/*w*) or 0.5% (*w*/*w*) garlic had better flavor, color, tenderness and overall acceptability, while juiciness increased with increasing garlic supplementation.

A final work worth mentioning is that authored by Quifer-Rada and colleagues [138]. In this experimental trial, the authors conducted an intervention study on pigs fed a standard diet for 3 days and then supplemented with a diet containing 1% (*w*/*w*) grape seed for 6 days. During the experimental trial, fecal samples were collected daily, and a combination of high-resolution non-targeted mass spectrometry, multivariate analysis (PLS-DA), data-dependent MS/MS scanning, and accurate mass database matching was performed. Targeted metabolomic analysis by GC-MS showed that the fecal excretion of cholesterol was increased by grape seed extract, resulting in decreased cholesterol in pork. In addition, intermediate metabolites of cholesterol biosynthesis, such as zymosterol, increased during the intervention, suggesting that endogenous cholesterol biosynthesis was stimulated due to decreased cholesterol absorption. The authors' innovative approach was the correlation between the observed biological effect with the main bioactive components contained in grape seeds after analytical identification. The authors hypothesized that the main agents responsible for the cholesterol-lowering action were both monomeric flavan-3-ols (catechins) and polymeric flavan-3-ols (proanthocyanidins) by increasing biliary excretion and reducing micellar solubility. Effectively, the hypocholesterolemic potential of catechins and other bioactive compounds derived from their condensation is well documented in humans. For example, Ngamukote et al. [141] demonstrated that gallic acid, catechin, and epicatechin have cholesterol-lowering activity by inhibiting pancreatic cholesterol esterase, binding bile acids, and reducing cholesterol solubility in micelles.

## 7. Conclusions

This review provided an overview of the reutilization of agro-food waste as potential ingredients to supplement the diets of farm pigs. Specifically, information on the number and type of publications, keywords, journals, countries, institutions, and trends were analyzed using VosViwer and BiblioShiny software. In particular, it has been shown that although America conducted research in this field between 2005 and 2010, today, it is Europe that leads this research topic. Most of the studies are in Environmental Sciences and Ecology, Agricultural Sciences, Energy and Fuels, Biotechnology and Chemistry, but most alarming is the low interest in Food Sciences and Veterinary Sciences, which should instead dominate among the research fields. In addition, the co-occurrence network map of terms used in the title, abstract, or as keywords provided insight to the hot topics addressed by countries over the years.

Regarding the use of agri-food waste to increase pig growth performance and decrease cholesterol levels in pork, forest plots have shown that their use can be seriously considered as a sustainable alternative to meet a zero-waste policy. Indeed, analyzing the studies involving plant-based waste in order to replace feed protein with a low-cost alternative, strong beneficial effects were reported. However, their efficacy seriously depends not only on the type of plant, duration of administration, or administration way, but especially on the inclusion rate within the feeds. Consequently, an experimental trial investigating dose–effect ratio is seriously recommended before potential application. Moreover, although many studies have demonstrated their efficacy as growth promoters and reducers of cholesterol levels in pork, only a few studies have investigated and demonstrated their influence on the overall health of pigs by evaluating physiological and histological parameters. Therefore, further studies on this aspect are also needed.

Looking ahead to future years and considering a global context in which agri-food waste is increasing, it is critical to direct scientific research toward circular economy approaches. However, the green strategy should actively involve not only farmers and companies, which should start applying greener methods to their production system, but also the modern consumers, who should instead be educated to recognize foods with low impact on the economy and the environment.

## Data Availability

No new data were created or analyzed in this study. Data sharing is not applicable to this article.

## Supplementary Materials

The following supporting information can be downloaded at: https://www.mdpi.com/article/10.3390/foods12030571/s1, Figure S1: World pork consumption. Darker colors indicate countries with higher pork consumption. Data were obtained from Statistica website (https://www.statista.com/, accessed on 10 January 2023)

## References

[1] Mannino G., Serio G., Gaglio R., Busetta G., La Rosa L., Lauria A., Settanni L., Gentile C. (2022). Phytochemical Profile and Antioxidant, Antiproliferative, and Antimicrobial Properties of Rubus idaeus Seed Powder. Foods.

[2] Gruber V., Holweg C., Teller C. (2016). What a waste! Exploring the human reality of food waste from the store manager’s perspective. J. Public Policy Mark..

[3] Lindgren E., Harris F., Dangour A.D., Gasparatos A., Hiramatsu M., Javadi F., Loken B., Murakami T., Scheelbeek P., Haines A. (2018). Sustainable food systems—A health perspective. Sustain. Sci..

[4] Agliassa C., Mannino G., Molino D., Cavalletto S., Contartese V., Bertea C.M., Secchi F. (2021). A new protein hydrolysate-based biostimulant applied by fertigation promotes relief from drought stress in *Capsicum annuum* L. Plant Physiol. Biochem..

[5] Anderson R., Bayer P.E., Edwards D. (2020). Climate change and the need for agricultural adaptation. Curr. Opin. Plant Biol..

[6] Rosenzweig C., Mbow C., Barioni L.G., Benton T.G., Herrero M., Krishnapillai M., Liwenga E.T., Pradhan P., Rivera-Ferre M.G., Sapkota T. (2020). Climate change responses benefit from a global food system approach. Nat. Food.

[7] Zhongming Z., Linong L., Xiaona Y., Wangqiang Z., Wei L. (2021). UNEP Food Waste Index Report 2021.

[8] Ferronato N., Torretta V. (2019). Waste mismanagement in developing countries: A review of global issues. Int. J. Environ. Res. Public Health.

[9] Farina V., Tinebra I., Perrone A., Sortino G., Palazzolo E., Mannino G., Gentile C. (2020). Physicochemical, nutraceutical and sensory traits of six papaya (*Carica papaya* L.) cultivars grown in greenhouse conditions in the Mediterranean climate. Agronomy.

[10] Capanoglu E., Nemli E., Tomas-Barberan F. (2022). Novel Approaches in the Valorization of Agricultural Wastes and Their Applications. J. Agric. Food Chem..

[11] Van Nguyen T.T., Phan A.N., Nguyen T.-A., Nguyen T.K., Nguyen S.T., Pugazhendhi A., Phuong H.H.K. (2022). Valorization of agriculture waste biomass as biochar: As first-rate biosorbent for remediation of contaminated soil. Chemosphere.

[12] Falade A.O. (2021). Valorization of agricultural wastes for production of biocatalysts of environmental significance: Towards a sustainable environment. Environ. Sustain..

[13] Garcia-Garcia G., Woolley E., Rahimifard S., Colwill J., White R., Needham L. (2017). A methodology for sustainable management of food waste. Waste Biomass Valorization.

[14] Tanveer U., Ishaq S., Gough A. (2021). Circular Economy in Agri-Food Sector: Food Waste Management Perspective. Challenges and Opportunities of Circular Economy in Agri-Food Sector.

[15] Bertocci F., Mannino G. (2022). Can Agri-Food Waste Be a Sustainable Alternative in Aquaculture? A Bibliometric and Meta-Analytic Study on Growth Performance, Innate Immune System, and Antioxidant Defenses. Foods.

[16] Parfitt J., Barthel M., Macnaughton S. (2010). Food waste within food supply chains: Quantification and potential for change to 2050. Philos. Trans. R. Soc. B Biol. Sci..

[17] Magangana T.P., Makunga N.P., Fawole O.A., Opara U.L. (2020). Processing factors affecting the phytochemical and nutritional properties of pomegranate (*Punica granatum* L.) peel waste: A review. Molecules.

[18] Martillanes S., Rocha-Pimienta J., Delgado-Adámez J. (2018). Agrifood by-products as a source of phytochemical compounds. Descriptive Food Science.

[19] Sharma A., Bachheti A., Sharma P., Bachheti R.K., Husen A. (2020). Phytochemistry, pharmacological activities, nanoparticle fabrication, commercial products and waste utilization of *Carica papaya* L.: A comprehensive review. Curr. Res. Biotechnol..

[20] Mannino G., Chinigò G., Serio G., Genova T., Gentile C., Munaron L., Bertea C.M. (2021). Proanthocyanidins and where to find them: A meta-analytic approach to investigate their chemistry, biosynthesis, distribution, and effect on human health. Antioxidants.

[21] Truong L., Morash D., Liu Y., King A. (2019). Food waste in animal feed with a focus on use for broilers. Int. J. Recycl. Org. Waste Agric..

[22] Read Q.D., Hondula K.L., Muth M.K. (2022). Biodiversity effects of food system sustainability actions from farm to fork. Proc. Natl. Acad. Sci. USA.

[23] Deselnicu D.C., Militāru G., Deselnicu V., Zăinescu G., Albu L. (2018). Towards a circular economy–a zero waste programme for Europe. Proceedings of the International Conference on Advanced Materials and Systems (ICAMS).

[24] Kass M.J. (2022). Climate, Sustainability, and Waste: EU and US Regulatory Approaches Compared. Interdisciplinary Approaches to Climate Change for Sustainable Growth.

[25] Bogusz M., Matysik-Pejas R., Krasnodębski A., Dziekański P. (2021). The concept of zero waste in the context of supporting environmental protection by consumers. Energies.

[26] Săplăcan Z., Márton B. (2019). Determinants of adopting a zero waste consumer lifestyle. Reg. Bus. Stud..

[27] Grigatti M., Barbanti L., Hassan M.U., Ciavatta C. (2020). Fertilizing potential and CO_2_ emissions following the utilization of fresh and composted food-waste anaerobic digestates. Sci. Total Environ..

[28] Chew K.W., Chia S.R., Yen H.-W., Nomanbhay S., Ho Y.-C., Show P.L. (2019). Transformation of biomass waste into sustainable organic fertilizers. Sustainability.

[29] Conrad Z., Niles M.T., Neher D.A., Roy E.D., Tichenor N.E., Jahns L. (2018). Relationship between food waste, diet quality, and environmental sustainability. PLoS ONE.

[30] Mannino G., Gentile C., Ertani A., Serio G., Bertea C.M. (2021). Anthocyanins: Biosynthesis, Distribution, Ecological Role, and Use of Biostimulants to Increase Their Content in Plant Foods—A Review. Agriculture.

[31] Campobenedetto C., Agliassa C., Mannino G., Vigliante I., Contartese V., Secchi F., Bertea C.M. (2021). A biostimulant based on seaweed (*Ascophyllum nodosum* and *Laminaria digitata*) and yeast extracts mitigates water stress effects on tomato (*Solanum lycopersicum* L.). Agriculture.

[32] Mapelli F., Carullo D., Farris S., Ferrante A., Bacenetti J., Ventura V., Frisio D., Borin S. (2022). Food waste-derived biomaterials enriched by biostimulant agents for sustainable horticultural practices: A possible circular solution. Front. Sustain..

[33] Xu L., Geelen D. (2018). Developing biostimulants from agro-food and industrial by-products. Front. Plant Sci..

[34] Galanakis C. (2020). Food waste valorization opportunities for different food industries. The Interaction of Food Industry and Environment.

[35] Durazzo A., Lucarini M., Heinrich M. (2022). Dietary Supplements, Botanicals and Herbs at the Interface of Food and Medicine. Front. Pharmacol..

[36] Spiker M.L., Hiza H.A.B., Siddiqi S.M., Neff R.A. (2017). Wasted food, wasted nutrients: Nutrient loss from wasted food in the United States and comparison to gaps in dietary intake. J. Acad. Nutr. Diet..

[37] Magara G., Prearo M., Vercelli C., Barbero R., Micera M., Botto A., Caimi C., Caldaroni B., Bertea C.M., Mannino G. (2022). Modulation of antioxidant defense in farmed rainbow trout (*Oncorhynchus mykiss*) fed with a diet supplemented by the waste derived from the supercritical fluid extraction of basil (*Ocimum basilicum*). Antioxidants.

[38] Mo W.Y., Cheng Z., Choi W.M., Lun C.H.I., Man Y.B., Wong J.T.F., Chen X.W., Lau S.C.K., Wong M.H. (2015). Use of food waste as fish feeds: Effects of prebiotic fibers (inulin and mannanoligosaccharide) on growth and non-specific immunity of grass carp (*Ctenopharyngodon idella*). Environ. Sci. Pollut. Res..

[39] Wong M.-H., Mo W.-Y., Choi W.-M., Cheng Z., Man Y.-B. (2016). Recycle food wastes into high quality fish feeds for safe and quality fish production. Environ. Pollut..

[40] Mechkirrou L., Arabi M., Ouhssine M., Afilal M.E.A. (2021). Food Waste reuse as a feed for organic chicken: A case study. Proceedings of the E3S Web of Conferences.

[41] Mechkirrou L., Ouhssine M., Afilal M.E.A. (2021). Valorisation of food waste as new raw materials in broiler feed. Proceedings of the E3S Web of Conferences.

[42] Salemdeeb R., Zu Ermgassen E.K.H.J., Kim M.H., Balmford A., Al-Tabbaa A. (2017). Environmental and health impacts of using food waste as animal feed: A comparative analysis of food waste management options. J. Clean. Prod..

[43] Westendorf M.L. (2000). Food waste as animal feed: An introduction. Food Waste to Animal Feed.

[44] Dou Z., Toth J.D., Westendorf M.L. (2018). Food waste for livestock feeding: Feasibility, safety, and sustainability implications. Glob. Food Sec..

[45] San Martin D., Ramos S., Zufía J. (2016). Valorisation of food waste to produce new raw materials for animal feed. Food Chem..

[46] Mateos-Aparicio I. (2021). Plant-based by-products. Food Waste Recovery.

[47] Minelgaitė A., Liobikienė G. (2019). Waste problem in European Union and its influence on waste management behaviours. Sci. Total Environ..

[48] Rauw W.M., Rydhmer L., Kyriazakis I., Øverland M., Gilbert H., Dekkers J.C.M., Hermesch S., Bouquet A., Gómez Izquierdo E., Louveau I. (2020). Prospects for sustainability of pig production in relation to climate change and novel feed resources. J. Sci. Food Agric..

[49] Van Eck N.J., Waltman L. (2011). Text mining and visualization using VOSviewer. arXiv.

[50] Srisusilawati P., Rusydiana A.S., Sanrego Y.D., Tubastuvi N. (2021). Biblioshiny R application on islamic microfinance research. Libr. Philos. Pract..

[51] Andrade C. (2020). Understanding the basics of meta-analysis and how to read a forest plot: As simple as it gets. J. Clin. Psychiatry.

[52] Hoffman J.R., Falvo M.J. (2004). Protein—Which is best?. J. Sport. Sci. Med..

[53] Berrazaga I., Micard V., Gueugneau M., Walrand S. (2019). The role of the anabolic properties of plant-versus animal-based protein sources in supporting muscle mass maintenance: A critical review. Nutrients.

[54] Fu Y., Therkildsen M., Aluko R.E., Lametsch R. (2019). Exploration of collagen recovered from animal by-products as a precursor of bioactive peptides: Successes and challenges. Crit. Rev. Food Sci. Nutr..

[55] Zhao X., Zhang X., Liu D. (2021). Collagen peptides and the related synthetic peptides: A review on improving skin health. J. Funct. Foods.

[56] Zhang Q., Hou Y., Bazer F.W., He W., Posey E.A., Wu G. (2021). Amino acids in swine nutrition and production. Amino Acids in Nutrition and Health.

[57] Chalvon-Demersay T., Luise D., Le Floc’H N., Tesseraud S., Lambert W., Bosi P., Trevisi P., Beaumont M., Corrent E. (2021). Functional amino acids in pigs and chickens: Implication for gut health. Front. Vet. Sci..

[58] Lebret B., Čandek-Potokar M. (2022). Pork quality attributes from farm to fork. Part I. Carcass and fresh meat. Animal.

[59] Rey A.I., Segura J.F., Castejón D., Fernández-Valle E., Cambero M.I., Calvo L. (2020). Vitamin D3 supplementation in drinking water prior to slaughter improves oxidative status, physiological stress, and quality of pork. Antioxidants.

[60] Uhlen M., Karlsson M.J., Zhong W., Tebani A., Pou C., Mikes J., Lakshmikanth T., Forsström B., Edfors F., Odeberg J. (2019). A genome-wide transcriptomic analysis of protein-coding genes in human blood cells. Science.

[61] Sjöstedt E., Zhong W., Fagerberg L., Karlsson M., Mitsios N., Adori C., Oksvold P., Edfors F., Limiszewska A., Hikmet F. (2020). An atlas of the protein-coding genes in the human, pig, and mouse brain. Science.

[62] Karlsson M., Sjöstedt E., Oksvold P., Sivertsson Å., Huang J., Álvez M.B., Arif M., Li X., Lin L., Yu J. (2022). Genome-wide annotation of protein-coding genes in pig. BMC Biol..

[63] Mannino G., Iovino P., Lauria A., Genova T., Asteggiano A., Notarbartolo M., Porcu A., Serio G., Chinigò G., Occhipinti A. (2021). Bioactive triterpenes of protium heptaphyllum gum resin extract display cholesterol-lowering potential. Int. J. Mol. Sci..

[64] Alsehli A.M., Liao S., Al-Sabri M.H., Vasionis L., Purohit A., Behare N., Clemensson L.E., Williams M.J., Schiöth H.B. (2022). The Statin Target HMG-Coenzyme a Reductase (Hmgcr) Regulates Sleep Homeostasis in Drosophila. Pharmaceuticals.

[65] Miyajima C., Hayakawa Y., Inoue Y., Nagasaka M., Hayashi H. (2022). HMG-CoA Reductase Inhibitor Statins Activate the Transcriptional Activity of p53 by Regulating the Expression of TAZ. Pharmaceuticals.

[66] Ding Y., Hou Y., Ling Z., Chen Q., Xu T., Liu L., Yu N., Ni W., Ding X., Zhang X. (2022). Identification of candidate genes and regulatory competitive endogenous RNA (ceRNA) networks underlying intramuscular fat content in yorkshire pigs with extreme fat deposition phenotypes. Int. J. Mol. Sci..

[67] Chalupová P., Sedláčková T., Kaplanová K., Weisz F., Bryndová M., Vykoukalová Z., Jůzl M., Šulcerová H., Gregor T., Urban T. (2012). Association of 15 candidate genes with meat quality traits in Czech Large White pigs. Afr. J. Agric. Res..

[68] Parmagnani A.S., Mannino G., Maffei M.E. (2022). Transcriptomics and Metabolomics of Reactive Oxygen Species Modulation in Near-Null Magnetic Field-Induced Arabidopsis thaliana. Biomolecules.

[69] Thewissen J.G.M., Cooper L.N., George J.C., Bajpai S. (2009). From land to water: The origin of whales, dolphins, and porpoises. Evol. Educ. Outreach.

[70] Sikorski Z.E., Kołakowska A., Pan B.S. (2020). The nutritive composition of the major groups of marine food organisms. Seafood: Resources, Nutritional Composition, and Preservation.

[71] Hamilton J.J., Auestad N., Innis S.M. (1994). A comparative study of hepatic HMG CoA reductase activity and LDL receptor relative mass in suckling and adult guinea pigs. Neonatology.

[72] West K.L., Luz Fernandez M. (2004). Guinea pigs as models to study the hypocholesterolemic effects of drugs. Cardiovasc. Drug Rev..

[73] Schoch L., Sutelman P., Suades R., Casani L., Padro T., Badimon L., Vilahur G. (2022). Hypercholesterolemia-induced HDL dysfunction can be reversed: The impact of diet and statin treatment in a preclinical animal model. Int. J. Mol. Sci..

[74] Gentile C., Mannino G., Palazzolo E., Gianguzzi G., Perrone A., Serio G., Farina V. (2020). Pomological, sensorial, nutritional and nutraceutical profile of seven cultivars of Cherimoya (*Annona cherimola Mill*.). Foods.

[75] Bodirsky B.L., Rolinski S., Biewald A., Weindl I., Popp A., Lotze-Campen H. (2015). Global food demand scenarios for the 21st century. PLoS ONE.

[76] Tripathi A.D., Mishra R., Maurya K.K., Singh R.B., Wilson D.W. (2019). Estimates for world population and global food availability for global health. The Role of Functional Food Security in Global Health.

[77] Lebret B., Čandek-Potokar M. (2022). Pork quality attributes from farm to fork. Part II. Processed pork products. Animal.

[78] Sooryanarain H., Meng X.-J. (2020). Swine hepatitis E virus: Cross-species infection, pork safety and chronic infection. Virus Res..

[79] Jennifer H., Yang L., Chen C., Fang J., Jin S., Li X., Su S., Wang W. (2022). Pig in the Middle: Environment, Health and Development Dimensions of the Pork Sector in China. Open J. Soc. Sci..

[80] Ge Y., Lin S., Li B., Yang Y., Tang X., Shi Y., Sun J., Le G. (2020). Oxidized pork induces oxidative stress and inflammation by altering gut microbiota in mice. Mol. Nutr. Food Res..

[81] Giromini C., Givens D.I. (2022). Benefits and Risks Associated with Meat Consumption during Key Life Processes and in Relation to the Risk of Chronic Diseases. Foods.

[82] Anihouvi D.G.H., Kpoclou Y.E., Assogba M.F., Iko Afé O.H., Lègba G., Scippo M., Hounhouigan D.J., Anihouvi V.B., Mahillon J. (2020). Microbial contamination associated with the processing of grilled pork, a ready-to-eat street food in Benin. J. Food Saf..

[83] Peruzy M.F., Houf K., Joossens M., Yu Z., Proroga Y.T.R., Murru N. (2021). Evaluation of microbial contamination of different pork carcass areas through culture-dependent and independent methods in small-scale slaughterhouses. Int. J. Food Microbiol..

[84] Lander B., Schneider M., Brunson K. (2020). A history of pigs in China: From curious omnivores to industrial pork. J. Asian Stud..

[85] Csonka A., Fertő I. (2020). Structural change and agglomeration in the Hungarian pork industry. Eur. Plan. Stud..

[86] Maes D.G.D., Dewulf J., Piñeiro C., Edwards S., Kyriazakis I. (2020). A critical reflection on intensive pork production with an emphasis on animal health and welfare. J. Anim. Sci..

[87] Shurson G.C. (2020). “What a waste”—Can we improve sustainability of food animal production systems by recycling food waste streams into animal feed in an era of health, climate, and economic crises?. Sustainability.

[88] Donthu N., Kumar S., Mukherjee D., Pandey N., Lim W.M. (2021). How to conduct a bibliometric analysis: An overview and guidelines. J. Bus. Res..

[89] Moral-Muñoz J.A., Herrera-Viedma E., Santisteban-Espejo A., Cobo M.J. (2020). Software tools for conducting bibliometric analysis in science: An up-to-date review. Prof. Inf..

[90] Shah S.H.H., Lei S., Ali M., Doronin D., Hussain S.T. (2019). Prosumption: Bibliometric analysis using HistCite and VOSviewer. Kybernetes.

[91] Xie L., Chen Z., Wang H., Zheng C., Jiang J. (2020). Bibliometric and visualized analysis of scientific publications on atlantoaxial spine surgery based on Web of Science and VOSviewer. World Neurosurg..

[92] Abafe E.A., Bahta Y.T., Jordaan H. (2022). Exploring Biblioshiny for Historical Assessment of Global Research on Sustainable Use of Water in Agriculture. Sustainability.

[93] Verhagen A.P., Ferreira M.L. (2014). Forest plots. J. Physiother..

[94] McDermott P.F., Zhao S., Wagner D.D., Simjee S., Walker R.D., White D.G. (2002). The food safety perspective of antibiotic resistance. Anim. Biotechnol..

[95] Vigliante I., Mannino G., Maffei M.E. (2019). OxiCyan^®^, a phytocomplex of bilberry (*Vaccinium myrtillus*) and spirulina (*Spirulina platensis*), exerts both direct antioxidant activity and modulation of ARE/Nrf2 pathway in HepG2 cells. J. Funct. Foods.

[96] Sagar N.A., Pareek S., Sharma S., Yahia E.M., Lobo M.G. (2018). Fruit and vegetable waste: Bioactive compounds, their extraction, and possible utilization. Compr. Rev. Food Sci. Food Saf..

[97] Mannino G., Gentile C., Maffei M.E. (2019). Chemical partitioning and DNA fingerprinting of some pistachio (*Pistacia vera* L.) varieties of different geographical origin. Phytochemistry.

[98] Mannino G., Maffei M.E. (2022). Metabolomics-Based Profiling, Antioxidant Power, and Uropathogenic Bacterial Anti-Adhesion Activity of SP4TM, a Formulation with a High Content of Type-A Proanthocyanidins. Antioxidants.

[99] Guimarães I., Baptista-Silva S., Pintado M., Oliveira A.L. (2021). Polyphenols: A promising avenue in therapeutic solutions for wound care. Appl. Sci..

[100] Yang Y., Zhang T. (2019). Antimicrobial activities of tea polyphenol on phytopathogens: A review. Molecules.

[101] Musarra-Pizzo M., Ginestra G., Smeriglio A., Pennisi R., Sciortino M.T., Mandalari G. (2019). The antimicrobial and antiviral activity of polyphenols from almond (*Prunus dulcis* L.) skin. Nutrients.

[102] Daglia M. (2012). Polyphenols as antimicrobial agents. Curr. Opin. Biotechnol..

[103] Othman L., Sleiman A., Abdel-Massih R.M. (2019). Antimicrobial activity of polyphenols and alkaloids in middle eastern plants. Front. Microbiol..

[104] Vigliante I., Mannino G., Maffei M.E. (2019). Chemical characterization and DNA fingerprinting of *Griffonia simplicifolia* baill. Molecules.

[105] Mhatre S., Srivastava T., Naik S., Patravale V. (2021). Antiviral activity of green tea and black tea polyphenols in prophylaxis and treatment of COVID-19: A review. Phytomedicine.

[106] Annunziata G., Sanduzzi Zamparelli M., Santoro C., Ciampaglia R., Stornaiuolo M., Tenore G.C., Sanduzzi A., Novellino E. (2020). May polyphenols have a role against coronavirus infection? An overview of in vitro evidence. Front. Med..

[107] Maffei M.E., Salata C., Gribaudo G. (2022). Tackling the Future Pandemics: Broad-Spectrum Antiviral Agents (BSAAs) Based on A-Type Proanthocyanidins. Molecules.

[108] Valenzuela-Grijalva N.V., Pinelli-Saavedra A., Muhlia-Almazan A., Domínguez-Díaz D., González-Ríos H. (2017). Dietary inclusion effects of phytochemicals as growth promoters in animal production. J. Anim. Sci. Technol..

[109] Coelho D., Pestana J., Almeida J.M., Alfaia C.M., Fontes C.M.G.A., Moreira O., Prates J.A.M. (2020). A high dietary incorporation level of *Chlorella vulgaris* improves the nutritional value of pork fat without impairing the performance of finishing pigs. Animals.

[110] Türkeli S., Kemp R., Huang B., Bleischwitz R., McDowall W. (2018). Circular economy scientific knowledge in the European Union and China: A bibliometric, network and survey analysis (2006–2016). J. Clean. Prod..

[111] Su G., Zhou X., Wang Y., Chen D., Chen G., Li Y., He J. (2018). Effects of plant essential oil supplementation on growth performance, immune function and antioxidant activities in weaned pigs. Lipids Health Dis..

[112] Devi S.M., Park J.W., Kim I.H. (2015). Effect of plant extracts on growth performance and insulin-like growth factor 1 secretion in growing pigs. Rev. Bras. Zootec..

[113] Liu Y., Che T.M., Song M., Lee J.J., Almeida J.A.S., Bravo D., Van Alstine W.G., Pettigrew J.E. (2013). Dietary plant extracts improve immune responses and growth efficiency of pigs experimentally infected with porcine reproductive and respiratory syndrome virus. J. Anim. Sci..

[114] Danilov A., Donică I., Coşman S., Savca D. (2018). Effectiveness of usage of cake obtained from grape seeds in the food of pigs for fattening. Proc. Zooteh. Şi Biotehnol. Agric..

[115] Yu Y., Xing Y., Li C., Wu X., Yang Z., Liu X., Zeng Q., Zhang B. (2018). Effects of linseed oil on growth performance, carcass traits and meat quality of Ningxiang pigs. Chin. J. Anim. Nutr..

[116] McNaughton E.P., Ball R.O., Friendship R.M. (1997). The effects of feeding a chocolate product on growth performance and meat quality of finishing swine. Can. J. Anim. Sci..

[117] Taranu I., Marin D.E., Palade M., Pistol G.C., Chedea V.S., Gras M.A., Rotar C. (2019). Assessment of the efficacy of a grape seed waste in counteracting the changes induced by aflatoxin B1 contaminated diet on performance, plasma, liver and intestinal tissues of pigs after weaning. Toxicon.

[118] Ogunsipe M.H., Ibidapo I., Oloruntola O.D., Agbede J.O. (2017). Growth performance of pigs on dietary cocoa bean shell meal. Livest. Res. Rural. Dev..

[119] Mabena P.M., Ratsaka M.M., Nkukwana T.T., Malebana I.M.M., Nkosi B.D. (2022). Growth performance, nutrient digestibility and carcass characteristics of pigs fed diets containing amarula (*Sclerocarya birrea* A. Rich) nut cake as replacement to soybean meal. Trop. Anim. Health Prod..

[120] Baruah K.K., Khargharia G., Deori S., Kadirvel G., Doley S., Baruah A., Abedin S.N., Sen A., Baruah Sr K.K. (2022). Effect of Dietary Substitution of Maize with Banana Pseudostem on Performance and Economics of Crossbred Grower Pigs. https://www.researchsquare.com/article/rs-1597153/v1.

[121] Liotta L., Chiofalo V., Lo Presti V., Chiofalo B. (2019). In vivo performances, carcass traits, and meat quality of pigs fed olive cake processing waste. Animals.

[122] Laitat M., Antoine N., Cabaraux J.-F., Cassart D., Mainil J., Moula N., Nicks B., Wavreille J., Philippe F.-X. (2015). Influence of sugar beet pulp on feeding behavior, growth performance, carcass quality and gut health of fattening pigs. Biotechnol. Agron. Société Environ..

[123] Badaras S., Klupsaite D., Ruzauskas M., Gruzauskas R., Zokaityte E., Starkute V., Mockus E., Klementaviciute J., Cernauskas D., Dauksiene A. (2022). Influence of Sugar Beet Pulp Supplementation on Pigs’ Health and Production Quality. Animals.

[124] Adebiyi O.A., Adeshola A.T., Ekeh C.C., Olumide M.D. (2020). Growth Performance, Digestibility and Gut Morphology of Grower Pigs fed Diets Substituted with Watermelon Waste. Anim. Nutr. Feed Technol..

[125] Giamouri E., Papadomichelakis G., Pappas A.C., Simitzis P.E., Galliou F., Paßlack N., Zentek J., Lasaridi K., Fegeros K., Manios T. (2022). Μeat Quality Traits as Affected by the Dietary Inclusion of Food Waste in Finishing Pigs. Sustainability.

[126] Md E.H., Seok Y.K., Chul J.Y. (2012). Dietary supplementation of green tea by-products on growth performance, meat quality, blood parameters and immunity in finishing pigs. J. Med. Plants Res..

[127] Pietrosemoli S., Moron-Fuenmayor O.E., Paez A., Villamide M.J. (2016). Effect of including sweet potato (*Ipomoea batatas Lam*.) meal in finishing pig diets on growth performance, carcass traits and pork quality. Anim. Sci. J..

[128] Olsson V., Pickova J. (2005). The influence of production systems on meat quality, with emphasis on pork. AMBIO A J. Hum. Environ..

[129] Cassens R.G. (2000). Historical perspectives and current aspects of pork meat quality in the USA. Food Chem..

[130] Son H.-Y., Lee M.-S., Chang E., Kim S.-Y., Kang B., Ko H., Kim I.-H., Zhong Q., Jo Y.-H., Kim C.-T. (2019). Formulation and characterization of quercetin-loaded oil in water nanoemulsion and evaluation of hypocholesterolemic activity in rats. Nutrients.

[131] Al Faraj G. (2020). Vegetal products with hypocholesterolemic activity. MedEspera.

[132] Pavlović N., Jokić S., Jakovljević M., Blažić M., Molnar M. (2020). Green extraction methods for active compounds from food waste—Cocoa bean shell. Foods.

[133] Oanh N.C., Lam T.Q., Tien N.D., Hornick J.-L., Ton V.D. (2021). Effects of medicinal plants mixture on growth performance, nutrient digestibility, blood profiles, and fecal microbiota in growing pigs. Vet. World.

[134] Ahmed S.T., Mun H.-S., Islam M.M., Ko S.-Y., Yang C.-J. (2016). Effects of dietary natural and fermented herb combination on growth performance, carcass traits and meat quality in grower-finisher pigs. Meat Sci..

[135] Serem J., Wahome R.G., Gakuya D., Onyango D.W. (2017). Growth performance, feed conversion efficiency and blood characteristics of growing pigs fed on different levels of *Moringa oleifera* leaf meal. J. Vet. Med. Anim. Health.

[136] Gunness P., Michiels J., Vanhaecke L., De Smet S., Kravchuk O., Van de Meene A., Gidley M.J. (2016). Reduction in circulating bile acid and restricted diffusion across the intestinal epithelium are associated with a decrease in blood cholesterol in the presence of oat β-glucan. FASEB J..

[137] Omojola A.B., Fagbuaro S.S., Ayeni A.A. (2009). Cholesterol content, physical and sensory properties of pork from pigs fed varying levels of dietary garlic (*Allium sativum*). World Appl. Sci. J..

[138] Quifer-Rada P., Choy Y.Y., Calvert C.C., Waterhouse A.L., Lamuela-Raventos R.M. (2016). Use of metabolomics and lipidomics to evaluate the hypocholestreolemic effect of Proanthocyanidins from grape seed in a pig model. Mol. Nutr. Food Res..

[139] Hanczakowska E., Świątkiewicz M., Grela E.R. (2017). Effect of dietary supplement of herbal extract from hop (*Humulus lupulus*) on pig performance and meat quality. Czech J. Anim. Sci..

[140] Taranu I., Habeanu M., Gras M.A., Pistol G.C., Lefter N., Palade M., Ropota M., Sanda Chedea V., Marin D.E. (2018). Assessment of the effect of grape seed cake inclusion in the diet of healthy fattening-finishing pigs. J. Anim. Physiol. Anim. Nutr..

[141] Ngamukote S., Mäkynen K., Thilawech T., Adisakwattana S. (2011). Cholesterol-lowering activity of the major polyphenols in grape seed. Molecules.

