# Supplementary material for: Pearls before Swine: Plant-Derived Wastes to Produce Low-Cholesterol Meat from Farmed Pigs—A Bibliometric Analysis Combined to Meta-Analytic Studies
Source: Foods. 2023 Jan 28;12(3):571. doi: 10.3390/foods12030571 (PMC9914002; doi:10.3390/foods12030571)
Supplement: Supplementary file 1 [file foods-12-00571-s001.zip › foods-2184107-supplementary.pdf]

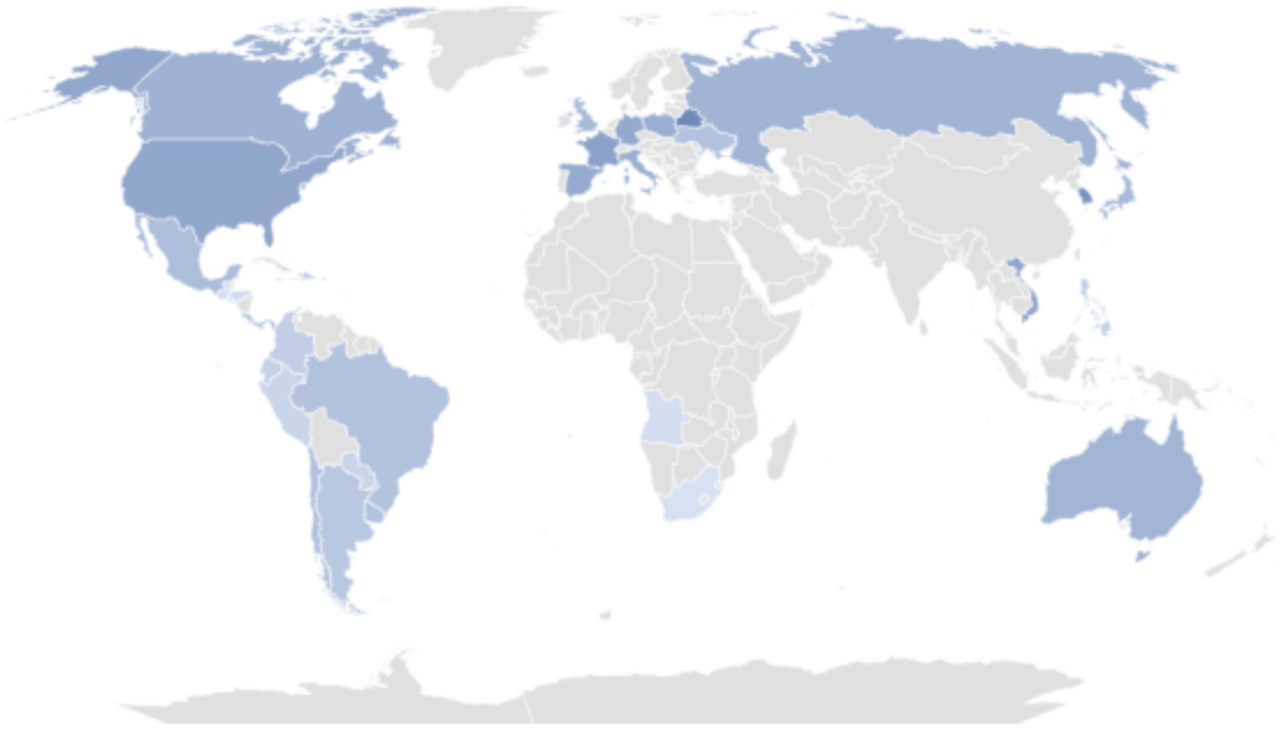

Figure S1. World pork consumption. Darker colors indicate countries with higher pork consumption. Data were obtained from Statistica website (https://www.statista.com/, accessed on 10 January 2023).
